# Difficulty in Swallowing

**Published:** 1903-11-07

**Authors:** 


### DIFFICULTY IN SWALLOWING.

In a clinical lecture delivered on this subject at St. Bartholomew's Hospital Mr. Butlin[1] classified cases according to the age at which the symptom occurred. He pointed out that up to the age of twenty chronic dysphagia is very rare. It may be due to some congenital malformation of the œsophagus or to tubercle of the pharynx or larynx, or to a cicatricial stricture caused by swallowing some caustic. In a child who was under his care and who had never been able to swallow solid food, the passage of a moderate-sized bougie, while the patient was under chloroform, effected a complete cure and enabled the child to take food in an ordinary manner. During the passage of the bougie some obstruction was felt near the middle of the œsophagus, but it was not sufficient to prevent the bougie being passed onwards into the stomach. Mr. Butlin believes that this was an instance of slight congenital malformation at a point where such malformations are known to occur.

Mr. Butlin then considers the period of life from twenty to forty, and here again chronic dysphagia is not common. In the first place malignant disease of the œsophagus is very rare before forty, although he mentions a case which occurred in a girl of twenty-four, which shows that it is unsafe to exclude the possibility of malignant stricture merely on the ground that the patient is too young. With regard to fibrous or cicatricial strictures, they are very rare. Spasmodic stricture may, however, occur at this period of life. After forty years of age chronic dysphagia becomes very much more common, and by far the most frequent cause is malignant disease of the œsophagus. There are many other possibilities; some patients have dilatation of the œsophagus, some have innocent strictures, some have diverticula, and some suffer from central or peripheral nervous disease. But all those things are rare, and out of every ten cases of difficulty in swallowing at this period of life it is probable that nine will be due to malignant disease of the œsophagus.

Mr. Butlin points out that it sometimes happens that although a good sized bougie can be passed into the stomach yet malignant disease of the œsophagus may be present, and in other instances where the bougie is arrested there may be no organic stricture present, the obstruction being merely due to spasm. Both of these facts are sources of mistakes and must be borne in mind. If there be any doubt about the existence of a spasmodic stricture it is a good plan to pass the bougie while the patient is under the influence of an anæsthetic, as by this means the spasm is prevented. In no case should an œsophageal bougie be passed until a most careful examination has been made of the patient with regard to the history, symptoms, and physical signs. The neck and chest should be examined in order to be sure there is no palpable tumour or undue fulness of the veins. The interior of the mouth should be examined in every part, and especially the base of the tongue where malignant disease is not by any means uncommon. The larynx should be inspected with the laryngoscope and the greatest care taken to observe if the vocal cords move readily. Paralysis of one vocal cord is a very important sign; it means generally one of two things: malignant disease of the œsophagus or aneurysm of the aorta. Paralysis or paresis of both cords may indicate disease of the central nervous system. Another important point which Mr. Butlin draws attention to is the significance of pain on swallowing or attempting to swallow in œsophageal stricture. Non-malignant strictures of the œsophagus do not as a rule give rise to pain in swallowing; and even malignant strictures, unless situated in the upper part, may exist for months or more than a year and yet never at any time cause the slightest pain in swallowing. On the other hand a spasmodic stricture sometimes causes very great pain.

Mr. Butlin lays special emphasis on the importance of exercising great caution in offering a diagnosis and prognosis of any case of chronic difficulty in swallowing in which the disease is beyond the reach of sight and touch. The evidence in such cases is for the most part circumstantial, and although mistakes are not often made yet it is always well to speak guardedly. To illustrate this he mentions the case of a patient in whom neither he nor other medical men could find evidence of organic stricture of the œsophagus, and in whom a bougie could apparently be passed with ease into the stomach, yet this patient died, in spite of successful gastrostomy, of wasting and exhaustion quite characteristic of malignant disease. In contrast to this was the case of a publican who suffered from chronic dysphagia. He had wasted rapidly, could only swallow liquids, and brought up large quantities of frothy mucus. A bougie was arrested low down in the œsophagus. A prognosis was made to the effect that the patient would be dead within six months. However, the patient did not die. After a while he began to grow better and in time completely recovered.

[1] Clinical Journal, Oct. 21.

